# Case Report: Retroperitoneal Laparoscopic Partial Nephrectomy for T2 Renal Cell Carcinoma During Pregnancy

**DOI:** 10.3389/fonc.2020.552228

**Published:** 2020-10-14

**Authors:** Jie Dong, Yi Zhao, Weifeng Xu

**Affiliations:** Urology Department of Peking Union Medical College Hospital, Beijing, China

**Keywords:** renal cell carcinoma, pregnancy, multidisciplinary team, retroperitoneal approach, laparoscopic partial nephrectomy

## Abstract

**Introduction:** Renal cell carcinoma (RCC) found during pregnancy is rare. Treatment strategies and timing of surgeries are controversial. Retroperitoneal laparoscopic partial nephrectomy for T2 RCC during pregnancy has not been reported before.

**Patient Concerns and Diagnosis:** Herein, we report a case of T2 RCC found in a 36-year-old woman during her 21st week of pregnancy. Both ultrasound and magnetic resonance imaging (MRI) suggested a malignancy, possibly renal cell carcinoma.

**Interventions and Outcomes:** After discussion with a multidisciplinary team, the tumor was removed completely via retroperitoneal laparoscopic partial nephrectomy, and pathology result was clear cell RCC. A male infant was delivered full-term uneventful, and both the patient and the boy were in good health after a 46-month follow-up.

**Conclusion:** Partial nephrectomy with retroperitoneal laparoscopic technique is feasible and recommended in some T2 RCC patients.

## Introduction

Renal cell carcinoma (RCC) is one of the common malignant tumors in the urinary system. Its pathological types include clear cell carcinoma, papillary cell carcinoma, and chromophobe cell carcinoma. During the early stage, there are no obvious clinical symptoms, and the prognosis is good after treatment, while in the late stage, patients may present with hematuria, lumbago, and abdominal mass, and the prognosis is usually poor.

Cancer diagnosis during pregnancy is a rare event. RCC diagnosed during gestation is extremely rare (1), which is first reported by Waddington et al. (2). Since then, only about 100 cases of this disease have been reported and, mostly, in the form of a case report (3–6). No standard treatment recommendations are available for these patients (7). Despite the fact that current guidelines for RCC recommend partial nephrectomy for all T1 RCCs and a portion of T2 RCCs, the majority of T1 or T2 RCC patients with pregnancy underwent radical nephrectomy. According to our limited knowledge, we herein report the first case of a T2 RCC patient who received retroperitoneal laparoscopic partial nephrectomy during gestation.

## Case

**Figure 1:**
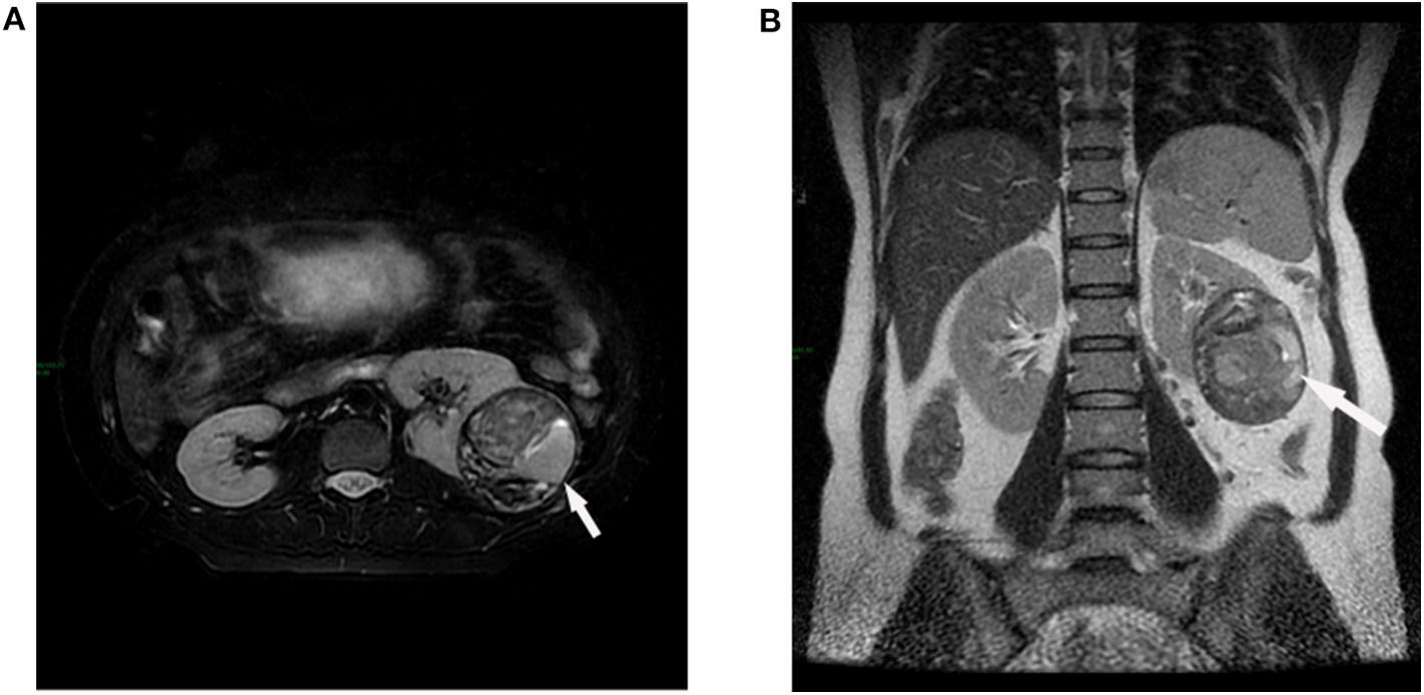
Abdominal magnetic resonance imaging (MRI). A round mass (white arrow) is shown in the middle part of the left kidney with a maximum diameter of 8.3 cm, heterogeneous signal with multiple partitions. **(A)** Axial plane. **(B)** Coronal plane.

A 36-year-old woman with 21 weeks of pregnancy was admitted to our hospital for a left renal tumor, which was incidentally detected by ultrasonography in a routine pregnancy examination. Ultrasound showed a confounding echo mass in the middle part of the left kidney, 7.9 cm in size, uneven internal echo, and clear blood flow signal inside. Computed tomography (CT) was not performed, as she was pregnant. Abdominal magnetic resonance imaging (MRI) showed a round mass in the middle part of the left kidney with a maximum diameter of 8.3 cm (Figure 1). The signal is heterogeneous, with some fluid visible inside the mass. Since angiomyolipoma usually shows hyperechoic rather than confounding echo in ultrasound, renal cancer was considered. The patient denied lower back pain, hematuria, fever, frequency of urination, urgency, pain, and other discomforts. Past medical history was unremarkable. The patient gave birth to a healthy girl 5 years ago. Personal history and family history were not remarkable. After admission, there were no abnormalities in vital signs, blood pressure was 130/80 mmHg, blood routine and biochemical examinations were within the normal range (hemoglobin was 114 g/L, serum creatinine was 47 μmol/L, and potassium was 3.7 mmol/L). Physical examination: There was no tenderness or muscle tension in the abdomen, and the mass was not touched.

A multidisciplinary team including urologists, gynecologists, pediatrists, anesthesiologists, and radiologists was responsible for the decision making to help the patient and her baby. Considering that the patient is only 36 years old, her strong desire to remove the tumor as well as retain the kidney, and the relatively indolent nature of the RCC, retroperitoneal laparoscopic partial nephrectomy was performed by an experienced surgeon after the risk was explained and informed consent was signed.

During the procedure, the patient was placed in the lateral flank position and underwent general anesthesia with endotracheal intubation. The procedure was performed through a retroperitoneal approach, and the retroperitoneal cavity was formed by blunt dissection and balloon dilation from a small incision located 2 cm above the iliac crest of the midaxillary line. After the establishment of the retroperitoneal space, four trocars were inserted on the left waist between the superior edge of the iliac spine and the inferior border of the rib. The tumor was removed completely, and renal reconstruction was then achieved with a 1/0 self-retaining barbed suture (V-Loc). Although maternal hemodynamic parameters were maintained stable and end-tidal CO_2_ was monitored below 35 mmHg, pneumoperitoneum pressure was strictly controlled below 12 mmHg to reduce maternal hypercapnia and fetal acidosis throughout the surgery. The operation time was 100 min with a warm ischemia time of 28 min and an estimated blood loss of 150 ml. The patient recovered uneventfully after the operation and was discharged within a week after the surgery. Blood hemoglobin was decreased postoperatively [95 g/L on postoperative day (POD) 1, 99 g/L on POD3] and back to normal on POD7 (121 g/L), while serum creatinine levels were normal throughout the perioperative period (66 μmol/L on POD1, 61 μmol/L on POD3, and 60 μmol/L on POD7). Obstetrics and gynecology consultation monitored the fetus before and throughout the operation.

**Figure 2:**
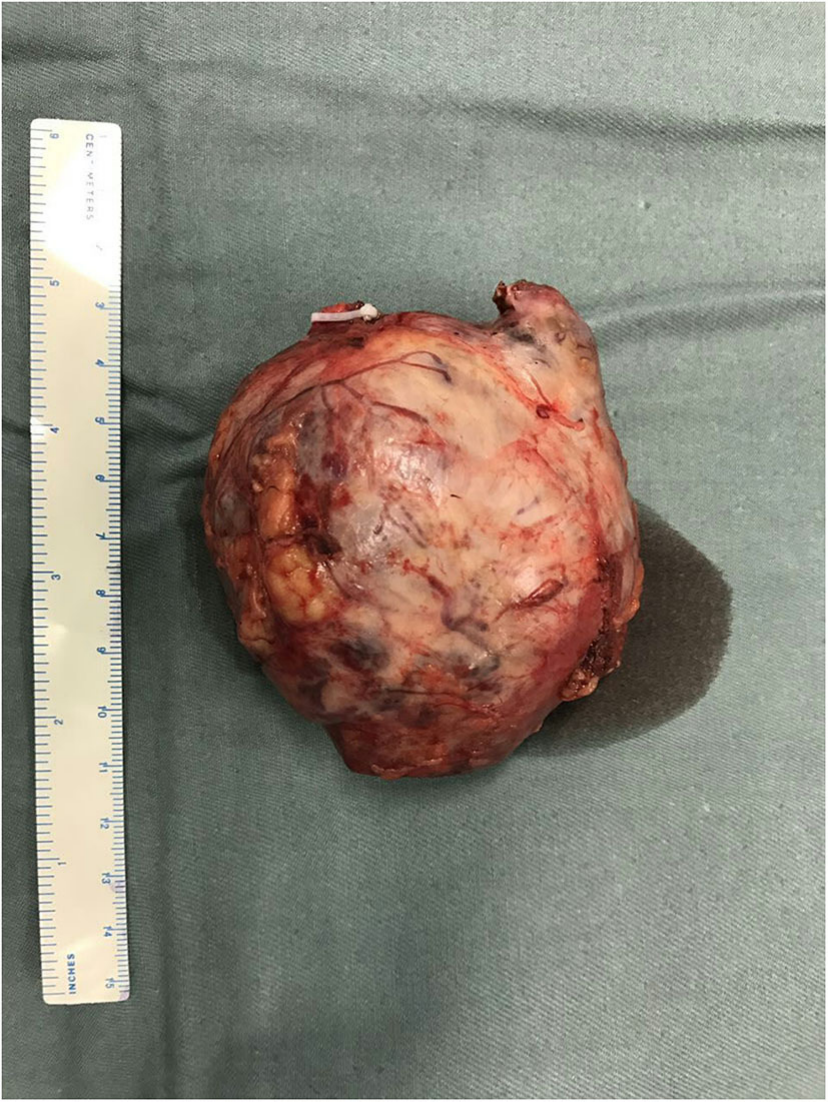
Gross section of the tumor. A solid tumor of 8 cm was removed, with a smooth capsule and a clear boundary with renal parenchyma.

The pathology report revealed an 8.2-cm clear RCC, Fuhrman grade 2, with negative surgical margins (Figure 2). According to the TNM classification system, it is classified as pT2aN0M0. During the 38th gestational week, a healthy male infant was born. We followed our patient every half year after surgery. Blood hemoglobin, serum creatinine, and thorax–abdominal–pelvic CT scan showed normochromic, normal renal function, and no sign of local recurrence or metastases. After 46 months of follow-up, the patient's baby is in good health and does not have any developmental birth defects. The whole treatment process is shown in Table 1.

**Table 1:** Timeline of the whole treatment process.

**Episode**	**Situation**
21st week of pregnancy	A T2 RCC was found
5 days after diagnosis	Surgery was performed
4th postoperative month	A healthy infant was born
every 6 month afterwards	Routine follow-up showed normochromic
46th postoperative month	The patient and the baby are doing fine

## Discussion

Cancer diagnosis during pregnancy is rare. The estimated incidence of cancer diagnosed during pregnancy in developed countries is 1 per 1,000 pregnancies (8). The most common tumors diagnosed in pregnant women are breast cancer, melanoma, cervical cancer, and lymphomas (8). RCC during gestation is extremely rare (1). However, renal cancer is the most common urologic tumor diagnosed during pregnancy (9). Some articles indicated that the increase in blood pressure (10) and estrogen level (11) during gestation might be related to RCC in a pregnant woman.

Most patients of this kind are asymptomatic, and renal tumors were found accidentally through routine ultrasound examination for antepartum care, which is the same as the case we reported. The most commonly reported symptoms include pain (50%), hematuria (47%), hypertension (18%), and the classical triad of hematuria, pain, and palpable mass (26%) (4). When a renal mass was suspected, and further imaging examination is needed, two factors should be taken into consideration: diagnostic accuracy and fetal safety. The safest imaging examinations during gestation are ultrasound and MRI, which do not affect the fetus. Ultrasound is sufficient in identifying renal masses, especially for those larger than 3 cm (12). Since tumor staging is one of the most important factors in decision making, MRI is a reproducible and a good, although expensive, method for the evaluation of renal lesions' size and location, their relationship with adjacent tissues, and possible cancer thrombus. Gadolinium, the contrast agent of MRI, may lead to fetal hypothyroidism (13). Thus, contrast MRI is not recommended in pregnant patients.

According to current studies, surgery is the best treatment for localized renal cancers. As a result of the improvements in surgical experience and technologies, most of the surgeries are being performed laparoscopically. Based on recent guidelines, partial nephrectomy has become the standard treatment for T1 renal cancers (14) and has proved to be feasible in some T2 renal cancers (15). However, lots of T1 and all of T2 renal cancers in pregnant women were treated by radical nephrectomy to shorten operation time and reduce perioperative complications (3, 5, 6). Here, we report the first case of a laparoscopic partial nephrectomy, applied for T2 RCC in a pregnant patient, which demonstrated the feasibility of this treatment, with a satisfactory outcome for both the patient and the fetus. We did this surgery through the retroperitoneal approach with the pneumoperitoneum pressure controlled below 12 mmHg during the 21st gestational week. Compared to the transperitoneal approach, the retroperitoneal approach, along with controlled pneumoperitoneum pressure, can reduce the impact of the abdominal pressure elevation and the acid–base balance disturbance caused by CO_2_ insufflation, which, in turn, jeopardized the blood flow and development of the fetus (5). When pneumoperitoneum pressure is strictly controlled, the operation space of the retroperitoneal approach is relatively small, which leads to the difficulty in operation. Therefore, we consider retroperitoneal laparoscopic partial nephrectomy as a safe treatment for pregnant women with T2 renal tumors depending on the surgeon's experience.

The timing of surgery remains controversial. When fetal maturity, and the volume and doubling time of RCC (normally 300–500 days) are considered (16), RCC diagnosed during the first trimester could go for surgical resection immediately with the consideration of abortion possibly, and for those diagnosed during the third trimester, some reports (3, 16) claimed that we could postpone the operation until the fetal lung matures or even after delivery, as long as the tumor is not rapidly growing. However, if diagnosed during the second trimester, as the case we reported, the decision would not be easy to make. Surgical intervention becomes difficult as the uterus enlarges, and manipulations may induce uterine contractions causing spontaneous abortion; at the same time, intraoperative blood loss can lead to hypotension and fetal hypoxia, which is harmful to the fetus (3). Therefore, if surgery is needed, a retroperitoneal approach, which we applied in this case, could be considered as a reliable choice because it can effectively avoid the disturbance of the abdominal cavity caused by the operation. However, some authors believe that the second trimester is the safest period for surgery (17). Consequently, there are no standardized guidelines for the management of this situation, and each case has its own particularity when making decisions. On the basis of respecting their wishes and focusing on fetal safety, it is important to have a thorough communication with patients, which helps them fully understand the potential benefits and risks of each treatment strategy.

To sum up, renal cancer in gestation is a rare clinical scenario. Most are found incidentally by routine pregnancy examination. Ultrasound and MRI are useful tools for identifying and staging the tumor. Though the timing is still controversial, nephrectomy remains a pivotal treatment for local RCC. Presently, partial nephrectomy is rarely applied, mostly through the transperitoneal approach, and only in pregnant patients with small tumors. This is the first presented retroperitoneal laparoscopic partial nephrectomy case in a pregnant patient with T2 RCC. Based on the multidisciplinary team's decision and surgeon's experience, partial nephrectomy can be performed retroperitoneally and securely in large-volume renal tumors in suitable pregnant patients.

The patient has provided informed consent for publication of the case.

## Data Availability Statement

All datasets generated for this study are included in the article/Supplementary Material.

## Ethics Statement

Written informed consent was obtained from the individual(s) for the publication of any potentially identifiable images or data included in this article.

## Author Contributions

JD, YZ, and WX collected clinical data. JD wrote the manuscript. WX revised the manuscript. All authors approved the submitted version.

## Conflict of Interest

The authors declare that the research was conducted in the absence of any commercial or financial relationships that could be construed as a potential conflict of interest.

## Abbreviations

RCC: renal cell carcinoma
MRI: magnetic resonance imaging.
